# Bayesian framework for analyzing adsorption processes observed via time-resolved X-ray diffraction

**DOI:** 10.1038/s41598-023-40573-z

**Published:** 2023-09-12

**Authors:** Yuichi Yokoyama, Shogo Kawaguchi, Masaichiro Mizumaki

**Affiliations:** 1 Japan Synchrotron Radiation Research Institute (JASRI), Sayo, Hyogo 679-5198 Japan; 2 Faculty of Science, Kumamoto University, Kurokami, Kumamoto, 860-8555 Japan

**Keywords:** Chemistry, Materials science, Mathematics and computing

## Abstract

Clarifying dynamic processes of materials is an important research topic in materials science. Time-resolved X-ray diffraction is a powerful technique for probing dynamic processes. To understand the dynamics, it is essential to analyze time-series data using appropriate time-evolution models and accurate start times of dynamic processes. However, conventional analyses based on non-linear least-squares fitting have difficulty both evaluating time-evolution models and estimating start times. Here, we establish a Bayesian framework including time-evolution models. We investigate an adsorption process, which is a representative dynamic process, and extract information about the time-evolution model and adsorption start time. The information enables us to estimate adsorption properties such as rate constants more accurately, thus achieving more precise understanding of dynamic adsorption processes. Our framework is highly versatile, can be applied to other dynamic processes such as chemical reactions, and is expected to be utilized in various areas of materials science.

## Introduction

In materials science, it is important to understand dynamic processes such as adsorptions, chemical reactions, and phase transitions for innovating materials and approaching practical applications. Since these dynamic processes generally involve changes in crystal structures and/or lattice constants, time-resolved X-ray diffraction (Tr-XRD) is a powerful probing technique. Tr-XRD enables the observation of continuous crystal structure changes^1–9^. In this study, we focus on the adsorption process of gas molecules in a metal–organic framework (MOF)^6–8^. It is important for innovating adsorption materials to analyze the adsorption/desorption dynamics of MOFs with appropriate time-evolution models and precise adsorption start times (${t}_{0}$). However, we have difficulties in evaluating time-evolution models and estimating ${t}_{0}$ based on conventional non-linear least-squares fitting because the information cannot be measured directly.

To overcome the difficulties, we utilize Bayesian inference for nonlinear regression. Bayesian inference is a method based on Bayes' theorem to estimate adsorption properties from experimental data as probability distributions under conditions where the experimental data have already been obtained. Conventional non-linear least-squares fitting has initial value dependence. In addition, non-linear least-squares fitting is possibly trapped in local minima and overfits to noise. However, Bayesian inference conquer these problems using the exchange Monte Carlo method^10,11^. These advantages become prominent in noisy data analysis because larger noise makes it more difficult for non-linear least-squares fitting to reach the global optimal solution. Since Bayesian inference estimates posterior probability distributions including information about both values and accuracies, Bayesian inference extracts more information from data than conventional non-linear least-squares fitting. In addition, the Bayesian model selection provides a significant advantage. In the analysis of multi-peak spectra, we have to determine the number of peaks prior to conventional non-linear least-squares fitting even though the actual number of peaks is often unknown. However, Bayesian inference enables the selection of the number of peaks among possible models^10,11^. Thus, Bayesian inference provides a superior solution^10–19^.

In this study, we incorporate time-evolution models, which describe the time evolution of peaks, into Bayesian inference and establish a Bayesian framework for analyzing dynamic processes observed via Tr-XRD. Our framework achieves the selection of the most plausible time-evolution model which is necessary to understand the dynamics. Our framework also enables the estimation of ${t}_{0}$ directly from Tr-XRD data on the basis of the time-evolution model. The information about the time-evolution model and ${t}_{0}$ enables us to understand the adsorption process accurately. We demonstrate the effectiveness of our framework by investigating a gas adsorption process.

## Results

### Data observation via time-resolved X-ray diffraction

We selected a typical MOF, a nanoporous Cu coordination polymer [{Cu_2_(pzdc)_2_(pyz)}_n_ (pzdc = pyrazine-2,3-dicarboxylate; pyz = pyrazine)] (CPL-1), as a sample. The CPL-1 with pillared-layer type MOF has one-dimensional nanochannels with dimensions of 4.0 Å × 6.0 Å^20^. This compound can adsorb various gases^21^, and the maximum entropy/Rietveld method revealed the crystal structure both without gas molecules and with adsorbed O_2_ gas^22^. Before experiments, we heated the CPL-1 sample at 373 K in a vacuum to remove adsorbed gas and H_2_O molecules. Then, we performed a static XRD measurement and confirmed that the XRD pattern was in good agreement with that in Refs 21, 22. The Ar gas adsorption process was observed via Tr-XRD at the BL02B2 beamline of SPring-8. The XRD patterns were measured continuously from $t=0$ with an exposure time of 0.333 s. In the middle of the measurement, we shot Ar gas molecules into the sample cell at $t=6.327 \mathrm{s} ({t}_{s})$. We focused on 031 reflection and analyzed the XRD patterns from 7.70° to 8.22°.

### Bayesian framework

First, we consider a generative model including a peak model and time-evolution models. The peak model determines the shape and number of diffraction peaks, while the time-evolution model expresses the time evolution of peak parameters such as peak areas and positions. By incorporating a time-evolution model, adsorption properties such as the rate constant $\left(\kappa \right)$, growth dimension $(n)$, and adsorption start time $({t}_{0}$) can replace peak parameters. This approach enables a more accurate and reliable analysis of dynamic adsorption processes, as it captures the continuous changes in crystal structures over time. We formulate the conditional probability of generated data $p({{\varvec{D}}}_{{\varvec{g}}{\varvec{e}}{\varvec{n}}}|\kappa ,n,{t}_{0},{\sigma }_{noise},\cdots )$ considering Gaussian noise with variance ${\sigma }_{noise}^{2}$. Then, applying Bayes' theorem to the conditional probability, we estimate the probability distribution of parameters conditioned on the observed data, i.e., the posterior probability distribution $p(\kappa ,n,{t}_{0},{\sigma }_{noise},\cdots |{\varvec{D}})$.

**Figure 1:**
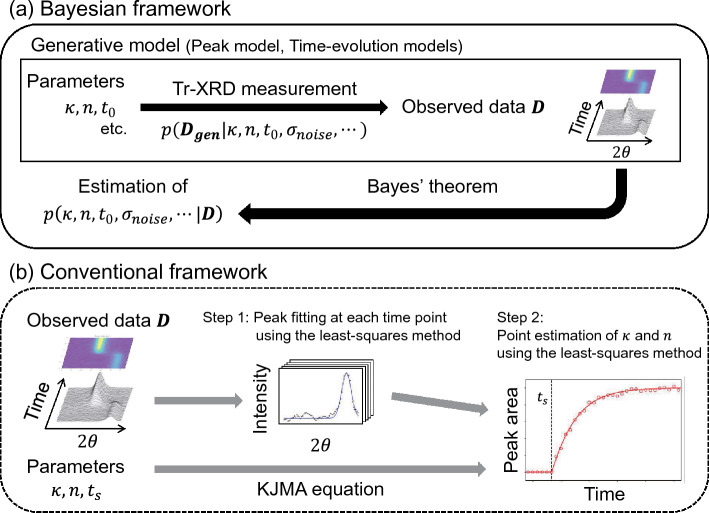
Difference between (**a**) the Bayesian framework and (**b**) the conventional framework for analyzing a dynamic adsorption process observed via time-resolved X-ray diffraction (Tr-XRD). (**a**) In the Bayesian framework, we construct the generative model including a peak model and time-evolution models. This is a probabilistic modeling of generated data conditioned on parameters and noise. Then, we use Bayes theorem to estimate the posterior probability distribution of adsorption properties such as the rate constant $\left(\kappa \right)$, growth dimension $(n)$, and adsorption start time $({t}_{0}$). (**b**) The conventional framework is based on the stepwise application of non-linear least-squares fitting, i.e. (Step 1) fitting the diffraction peaks at each time point and (Step 2) fitting the variation in the peak area over time with the Kolmogorov–Johnson–Mehl–Avrami (KJMA) equation and gas-shot time (${t}_{s}$).

Comparison between the Bayesian framework and the conventional framework is shown in Fig. 1. The conventional framework is based on the stepwise application of non-linear least-squares fitting, i.e., (Step 1) fitting the diffraction peaks at each time point and (Step 2) fitting the variation in the peak area over time with the Kolmogorov–Johnson–Mehl–Avrami (KJMA) equation^23,24^. The conventional framework corresponds to point estimation without considering the accuracy of estimation. In contrast, the Bayesian framework enables the estimation of the posterior probability distribution, thereby providing more information based on the shape and width of the distribution. In addition, the Bayesian framework realizes the estimation of ${t}_{0}$ directly from the observed data, while the conventional framework has difficulty extracting information about ${t}_{0}$ from data and requires the gas-shot time (${t}_{s}$). The Bayesian framework also allows the most plausible model to be selected from possible time-evolution models using Bayes free energy, which is one of information criteria used to evaluate the consistency between data and models in Bayesian statistics^10,11^.

### Bayesian model selection

In the Bayesian analysis, estimations based on different analysis models were performed to select the most plausible model. We adopted the peak model with two Gaussian functions corresponding to the adsorption and desorption phase, while we designed two different time-evolution models, i.e., Model 1 ($M=1$) and Model 2 ($M=2$). For both models, the time evolution of the peak area was assumed to follow the KJMA equation, as in previous studies^6–8^. In this study, it is assumed that the adsorption dynamics observed by Tr-XRD can be explained by the KJMA equation. Although time variation of the average structure is observed in Tr-XRD measurements, we attempted to estimate ${t}_{0}$ from Tr-XRD data. The time evolution of the peak center angle was assumed to be linear, given that there is little angular variation in each phase. On the other hand, the time evolution of the peak full width at half maximum (FWHM) was assumed to be constant in Model 1 and exponential in Model 2, indicating the absence and presence of peak broadening in the adsorption dynamics. To perform the analysis without bias, the prior probabilities of the models were set as $p\left(M=1\right)=p\left(M=2\right)=0.5$, and the prior probability distribution of each parameter was set to be uniform. For obtaining the posterior probability distribution, we used the exchange Monte Carlo method. In this method, the Monte Carlo sampling in each replica with each virtual temperature and exchanges of samples between neighboring replicas enable escape from local optima and efficient achievement of the global optimal solution. By utilizing sampling results in all replicas, it is also possible to calculate Bayes free energy. The total number of the Monte Carlo sweeps was 10,000 including 5,000 burn-in sweeps.

**Figure 2:**
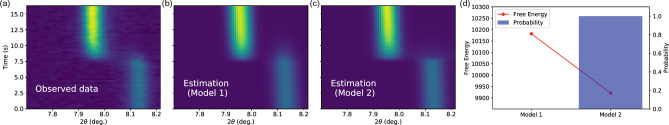
(**a**–**c**) Color maps of the observed time-resolved X-ray diffraction patterns and estimated results with Model 1 and Model 2. (**d**) Comparison between Bayes free energies of Model 1 and Model 2. The model selection probabilities are also shown.

Figure 2a–c shows the color maps of the observed Tr-XRD patterns and estimated results with Model 1 and Model 2. Both the models appear to be in good qualitative agreement with the observed data. To calculate the model selection probabilities $p\left(M|{\varvec{D}}\right)$, which quantify the realization probability of each model, we compared Bayes free energies in Model 1 and Model 2. Bayes free energy of Model 2 is lower than that of Model 1 by around 300, meaning that $p\left(M=2|{\varvec{D}}\right)$ is almost equal to 1 as shown in Fig. 2d. The Bayesian model selection suggests that Model 2 is much better than Model 1, which indicates the peak broadening during the adsorption process.

### Estimation of the adsorption properties

**Figure 3:**
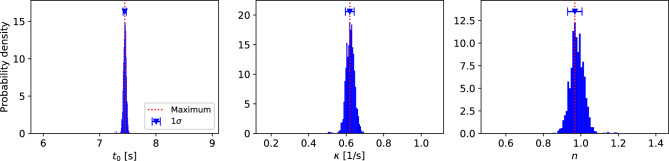
Marginalized posterior probability distributions of the adsorption properties, i.e., the adsorption start time (${t}_{0}$), rate constant ($\kappa$), and number of dimensions ($n$). The vertical line and error bar for each parameter correspond to the maximum and one standard deviation ($1\sigma$) of the distribution.

Based on the selected model (Model 2), we estimated the adsorption properties using the posterior probability distribution. Since $p\left({{\varvec{\theta}}}_{M=2},\sigma |{\varvec{D}},M=2\right)$ is a multivariate probability distribution and difficult to visualize, we consider the marginalized posterior probability distributions $p\left({\theta }_{M=2}^{k}|{\varvec{D}},M=2\right)=\iint p\left({{\varvec{\theta}}}_{M=2},\sigma |{\varvec{D}},M=2\right)d{{\varvec{\theta}}}_{M=2}^{\neg k}d\sigma$, where $k$ denotes the index of a specific parameter. By using the marginalization, we can obtain the one-dimensional probability distribution of each parameter. Figure 3 shows the marginalized posterior probability distributions of the adsorption properties, i.e., the adsorption start time (${t}_{0}$), rate constant ($\kappa$), and number of dimensions ($n$). Each probability distribution in Fig. 3 consists of a single peak, indicating that the optimal value of each parameter was uniquely estimated. By using one standard deviation ($1\sigma$) of each distribution as a measure of accuracy, the adsorption start time was estimated to be ${t}_{0}=7.4461\pm 0.0287$ s. The estimated value of ${t}_{0}$ deviates significantly from ${t}_{s}=6.327 \mathrm{s}$, indicating that the time lag between ${t}_{s}$ and ${t}_{0}$ was large in the present experiment. Since the conventional analysis substitutes ${t}_{s}$ for ${t}_{0}$, the large time lag leads to misunderstanding of the dynamics. Since ${t}_{s}$ is usually selected from the time points observed every 0.333 s, the accuracy of ${t}_{0}$ in the Bayesian analysis (~ 0.029 s) is one order of magnitude greater than that in the conventional analysis (~ 0.333 s). The rate constant and the number of dimensions were estimated to be $\kappa =0.6192\pm 0.0235$ 1/s and $n=0.9680\pm 0.0382$ in the Bayesian analysis. On the other hand, in the conventional analysis, the values were estimated to be $\kappa =0.1535$ 1/s and $n=1.8460$. We consider that these differences are primarily due to the large time lag between ${t}_{gate}$ and ${t}_{0}$.

**Figure 4:**
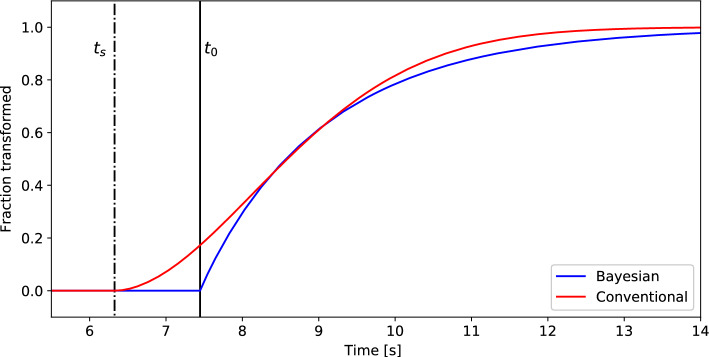
Fractions of the adsorption phase estimated by the Bayesian framework and the conventional framework. The blue line denotes the Bayesian analysis result, while the red line corresponds to the conventional result. The vertical lines represent the gas-shot time (${t}_{s}$) and estimated adsorption start time (${t}_{0}$).

To visualize the difference in results between the Bayesian analysis and the conventional analysis, we employed the time evolution of fractions transformed to the adsorption phase. The time evolution of the fractions refers to 031 diffraction peak area of the adsorption phase and was estimated by using the KJMA equation. Figure 4 shows the comparison of the fractions estimated by the Bayesian analysis and the conventional analysis. As shown in the figure, the time evolution of the Bayesian framework is apparently different from that of the conventional framework. Since the difference is significant in the time region between ${t}_{s}$ and ${t}_{0}$, estimating ${t}_{0}$ is quite important for understanding the dynamics. To examine the accuracy of the estimated ${t}_{0}$, we consider that a comparison with another probing technique such as X-ray absorption spectroscopy would be helpful because XRD is inherently less sensitive to local phenomena.

### Validation of the Bayesian analysis with numerical simulations

**Figure 5:**
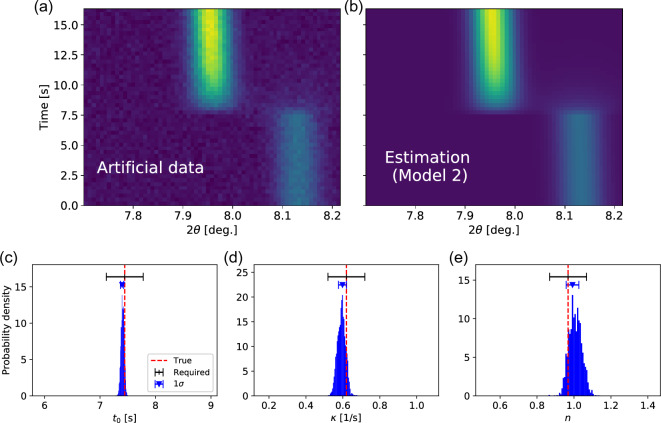
Color maps of the (**a**) artificial data and (**b**) estimation by the Bayesian framework. Posterior probability distributions of the (**c**) adsorption start time, (**d**) rate constant, and (**e**) number of dimensions. The vertical dashed lines denote the true values. The error bars for each parameter correspond to the accuracy required to understand the dynamics and one standard deviation ($1\sigma$) of the distribution.

To validate deviation between true values and estimated values in the Bayesian framework, we performed numerical simulations using artificial Tr-XRD data. The artificial data was generated based on Model 2 by adding Gaussian noise with the same level as the observed data. The Bayesian analysis of the artificial data was performed by using Model 2 under the same prior distributions as the observed data analysis. The color maps of the artificial data and the estimation results are shown in Fig. 5a,b. These color maps are in good qualitative agreement, indicating successful fitting of the artificial data.

Figure 5c–e shows the marginalized posterior probability distributions of the adsorption properties. The adsorption start time was estimated to be ${t}_{0}=7.3982\pm 0.0298$ s, while the true value was ${t}_{0}=7.4461$ s. The true value is outside of the estimated ${t}_{0}$ range (from 7.3684 s to 7.4280 s). However, the true value is included in the probability distribution and the estimation accuracy is much higher than the conventionally required accuracy (~ 0.333 s) as shown in Fig. 5c. Therefore, the estimation of ${t}_{0}$ is accurate enough to understand the adsorption dynamics. The rate constant and number of dimensions were estimated to be $\kappa =0.5980\pm 0.0219$ 1/s and $n=0.9921\pm 0.0343$, respectively. Since the true values were $\kappa =0.6192$ 1/s and $n=0.9680$, the values are included in the estimated ranges of $\kappa$ and $n$. In addition, the accuracies required to understand the dynamics are ~ 0.1 s and ~ 0.1 in the present case, indicating that the estimation of $\kappa$ and $n$ was successfully performed. The results of the numerical simulations show that the information about the adsorption properties can be extracted by the Bayesian analysis with high accuracy even when the data contains considerable noise, indicating that the Bayesian analysis enables precise understanding of the dynamics from experimentally observed data.

## Discussion

To understand the dynamics observed via Tr-XRD, it is essential to analyze the data using the appropriate time-evolution model ($M$) and precise start time (${t}_{0}$). Since $M$ and ${t}_{0}$ cannot be measured directly, the conventional analysis has difficulty extracting the information about $M$ and ${t}_{0}$ from the experimentally observed data. To overcome the difficulties, we established the Bayesian framework for analyzing dynamic processes and demonstrated the Bayesian analysis of the Ar gas adsorption process in a typical MOF.

The Bayesian model selection suggested that Model 2 was much better than Model 1, indicating the peak broadening during the adsorption process. Although human judgement has been used to determine the time evolution such as the peak broadening and shift, the Bayesian analysis enables the selection of the most plausible time-evolution model without involving human judgement.

Since the conventional analysis substitutes ${t}_{s}$ for ${t}_{0}$, we understood the adsorption dynamics incorrectly due to the large time lag between ${t}_{s}$ and ${t}_{0}$. However, the Bayesian analysis enables the estimation of ${t}_{0}$ directly from the experimental data with an accuracy one order of magnitude greater than that of the conventional analysis. In addition, other adsorption properties such as the rate constant ($\kappa$) and growth dimension ($n$) were estimated with much higher accuracies than the accuracies required to understand the dynamics.

The estimated rate constant of Ar gas adsorption was lower than that of O_2_ gas adsorption reported in ref.6. If the time difference between ${t}_{s}$ and ${t}_{0}$ is small in the O_2_ gas adsorption measurements, the slower adsorption can be attributed to the stronger influence of the potential barrier in the nanopores because the molecular size of Ar (3.405 Å) is larger than that of O_2_ (2.930 Å)^6,25^. The number of dimensions was estimated to be ~ 1, indicating quasi-one-dimensional growth in the Ar gas adsorption phase, which is similar to the O_2_ gas adsorption case. By applying the Bayesian analysis to various gas adsorption processes, more quantitative comparison can be realized on the basis of the posterior probability distributions. We also consider that the integrated Bayesian analysis of multiple diffraction peaks is effective for deeper understanding of the adsorption dynamics.

Our framework can be applied to other dynamic processes by modifying the time-evolution models. We should note that the applications are limited to dynamic processes for which time-evolution models can be formulated. For example, in the case of solid-state chemical reactions, various reaction models such as nucleation models and geometrical contraction models can be used to represent the time evolution^26^. The Bayesian analysis enables the selection of the most plausible reaction model among existing reaction models. It may be also possible to design new models and evaluate the models through the Bayesian analysis, which could lead to advances in reaction model research. Moreover, our framework can be applied to a wide variety of one-dimensional datasets observed by various measurements such as electron powder diffraction, neutron powder diffraction, and various types of spectroscopies. It is also possible in principle to extend our framework to two-dimensional datasets. Hence, the Bayesian analysis is expected to be utilized in various research areas of materials science.

## Methods

### Time-resolved X-ray diffraction measurement

The CPL-1 sample was placed at 2 mm from the tip into a borosilicate capillary with a 0.5-mm inner diameter, which was attached to a stainless-steel tube with double O-rings. Time-resolved in situ synchrotron X-ray powder diffraction patterns of CPL-1 during adsorption of Ar at 100 K were measured on the BL02B2 beamline of the SPring-8 synchrotron facility, Japan, by using a large Debye–Scherrer-type diffractometer with a multi-modular system constructed with six MYTHEN detectors^27^. The sample temperature was controlled by hot N_2_ gas flow devices, and the sample atmosphere was controlled with a remote gas- and vapor-pressure control (RGVPC) system^5^. The data were collected with a flat panel detector linked to the RGVPC system over the $2\theta$ range from 1° to 30°. We converted the observed two-dimensional diffraction patterns to one-dimensional patterns using pyFAI library^28^.

The energy of the incident X-ray was set to 15.5 keV. The X-ray diffraction patterns were continuously obtained by exposing the sample for 0.333 s. For short periods of time, measurement cycles and gas pressure monitoring cycles coincide on the order of 10 ms. At 6.327 s after starting the measurements, Ar gas at 41 kPa filled in a gas manifold of a remote gas handling system was automatically introduced into the glass capillary in which the CPL-1 sample was maintained in a vacuum. The Ar gas pressure was decreased to 20 kPa within 1 s at the latest and the pressure was rigorously kept constant during the measurements.

### Generative model for time-resolved X-ray diffraction patterns

The time-resolved X-ray diffraction data are given as ${\varvec{D}}=\left\{{\varvec{x}},{\varvec{t}},{\varvec{I}}\right\}={\left\{{x}_{i},{t}_{j},{I}_{ij}\right\}}_{i=1,\dots {,N}_{x},j=1,\dots ,{N}_{t}}$, where ${\varvec{x}}$, ${\varvec{t}}$, and ${\varvec{I}}$ denote the diffraction angles, time points, and diffraction intensities, respectively. We consider that a diffraction intensity at an angle ${x}_{i}$ and a time point ${t}_{j}$ is generated by adding measurement noise ${\varepsilon }_{ij}$ as

1 $${I}_{ij}=f\left({x}_{i},{t}_{j};{{\varvec{\theta}}}_{M},M\right)+{\varepsilon }_{ij},$$

where $f\left({x}_{i},{t}_{j};{{\varvec{\theta}}}_{M},M\right)$ is a model function with parameters ${{\varvec{\theta}}}_{M}$ and $M$. If the noise is assumed to follow a Gaussian distribution as ${\varepsilon }_{ij}\sim \mathcal{N}(0,{\sigma }^{2})$ with noise variance ${\sigma }^{2}$, the conditional probability $p\left({I}_{ij}|{{\varvec{\theta}}}_{M},M,\sigma ,{x}_{i},{t}_{j}\right)$ is equal to $p({\varepsilon }_{ij})$ and is represented as

2 $$p\left({I}_{ij}|{{\varvec{\theta}}}_{M},M,\sigma ,{x}_{i},{t}_{j}\right)=\frac{1}{\sqrt{2\pi {\sigma }^{2}}}\mathrm{exp}\left[-\frac{{\left\{{I}_{ij}-f\left({x}_{i},{t}_{j};{{\varvec{\theta}}}_{M},M\right)\right\}}^{2}}{2{\sigma }^{2}}\right].$$

Regarding the noise at each data point as independent, we can express the conditional probability for all data points as

3 $$p\left({\varvec{I}}|{{\varvec{\theta}}}_{M},M,\sigma ,{\varvec{x}},{\varvec{t}}\right)={\prod }_{i=1}^{{N}_{x}}{\prod }_{j=1}^{{N}_{t}}p\left({I}_{ij}|{{\varvec{\theta}}}_{M},M,\sigma ,{x}_{i},{t}_{j}\right)=\mathrm{exp}\left\{-{N}_{x}{N}_{t}E\left({{\varvec{\theta}}}_{M},M,\sigma \right)\right\},$$

where

4 $$E\left({{\varvec{\theta}}}_{M},M,\sigma \right)\equiv \frac{1}{2{N}_{x}{N}_{t}{\sigma }^{2}}{\sum }_{i=1}^{{N}_{x}}{\sum }_{j=1}^{{N}_{t}}{\left\{{I}_{ij}-f\left({x}_{i},{t}_{j};{{\varvec{\theta}}}_{M},M\right)\right\}}^{2}+\frac{\mathrm{ln}\left(2\pi {\sigma }^{2}\right)}{2}.$$

In a typical time-resolved X-ray diffraction experiment, ${\varvec{x}}$ and ${\varvec{t}}$ are set before the measurements in a non-probabilistic manner, which means $p\left({\varvec{D}}|{{\varvec{\theta}}}_{M},M,\sigma \right)=p\left({\varvec{I}}|{{\varvec{\theta}}}_{M},M,\sigma ,{\varvec{x}},{\varvec{t}}\right)$.

### Model functions for recognizing the observed data

Assuming the shape of a diffraction peak to be a Gaussian function, we can express the model function $f\left(x,t;{{\varvec{\theta}}}_{M},M\right)$ as

5 $$f\left(x,t;{{\varvec{\theta}}}_{M},M\right)={\sum }_{i=1}^{2}G\left\{x,t;{a}_{i}^{M}\left(t\right),{c}_{i}^{M}\left(t\right),{w}_{i}^{M}\left(t\right) \right\},$$

where

6 $$G\left\{x,t;{a}_{i}^{M}\left(t\right),{c}_{i}^{M}\left(t\right),{w}_{i}^{M}\left(t\right) \right\}=\frac{\sqrt{4\mathrm{ln}2}}{\sqrt{\pi }}\frac{{a}_{i}^{M}\left(t\right)}{{w}_{i}^{M}\left(t\right)}\mathrm{exp}\left[-\frac{4\mathrm{ln}2{\left\{x-{c}_{i}^{M}\left(t\right)\right\}}^{2}}{{\left\{{w}_{i}^{M}\left(t\right)\right\}}^{2}}\right].$$

Here, $i=1$ and $i=2$ correspond to the adsorption and desorption phases, respectively. ${a}_{i}^{M}\left(t\right)$, ${c}_{i}^{M}\left(t\right)$, and ${w}_{i}^{M}\left(t\right)$ represent the time-evolution function for the peak area, center angle, and full width at half maximum (FWHM), respectively. In this study, we designed two different time-evolution models, i.e., Model 1 ($M=1$) and Model 2 ($M=2$), and selected a more plausible model using Bayes free energy, as described below. In both models, we used the Kolmogorov–Johnson–Mehl–Avrami equation^23,24^ for the time-evolution of the peak area as

7 $$ a_{1}^{M} \left( t \right) = \left\{ {\begin{array}{*{20}c}    {A\left[ {1 - {\text{exp}}\left\{ { - \kappa \left( {t - t_{0} } \right)^{n} } \right\}} \right]} & {t \ge t_{0} }  \\    0 & {t < t_{0} }  \\   \end{array} } \right. $$

and

8 $$   a_{2}^{M} \left( t \right) = \left\{ {\begin{array}{*{20}c}    {B{\text{exp}}\left\{ { - \kappa \left( {t - t_{0} } \right)^{n} } \right\}} & {t \ge t_{0} }  \\    B & {t < t_{0} }  \\   \end{array} ,} \right. $$

where ${t}_{0}$, $\kappa$, and $n$ indicate the adsorption start time, rate constant, and number of dimensions, respectively, while $A$ and $B$ correspond to the scale parameters. For the time evolution of the peak center angles, assuming little or no angular variation, we used linear functions as

9 $${c}_{1}^{M}\left(t\right)=Ct+D$$

and

10 $${c}_{2}^{M}\left(t\right)=Et+F$$

with the parameters $C$, $D$, $E$, and $F$.

On the other hand, we considered two different time-evolution functions for the FWHM. In Model 1, assuming a constant width during the measurement, we used constant values of ${w}_{1}^{M=1}\left(t\right)=G$ and ${w}_{2}^{M=1}\left(t\right)=H$. In Model 2, assuming an increase in the peak width during the adsorption process, we designed the time-evolution functions using the parameters $G$, $H$, $I$, and $J$ as

11 $$ w_{1}^{{M = 2}} \left( t \right) = \left\{ {\begin{array}{*{20}c}    {G{\text{exp}}\left\{ { - \kappa \left( {t - t_{0} } \right)} \right\} + H} & {t \ge t_{0} }  \\    {G + H} & {t < t_{0} }  \\   \end{array} } \right.. $$

and

12 $$ w_{2}^{{M = 2}} \left( t \right) = \left\{ {\begin{array}{*{20}c}    {I\left[ {1 - {\text{exp}}\left\{ { - \kappa \left( {t - t_{0} } \right)} \right\}} \right] + J} & {t \ge t_{0} }  \\    J & {t < t_{0} }  \\   \end{array} } \right.. $$

The parameter set in Model 1 is represented as ${{\varvec{\theta}}}_{M=1}=\left\{{t}_{0},\kappa ,n,A,B,C,D,E,F,G,H\right\}$, while that in Model 2 is modified to ${{\varvec{\theta}}}_{M=2}=\left\{{t}_{0},\kappa ,n,A,B,C,D,E,F,G,H,I,J\right\}$.

### Generation of artificial Tr-XRD data

We generated artificial data based on the generative model and the model functions with Model 2 using parameters shown in Table 1. We used random numbers to generate noise.

**Table 1:** Parameters used for generating artificial Tr-XRD data.

${t}_{0}$	$\kappa$	$n$	$A$	$B$	$C$	$D$	$E$	$F$	$G$	$H$	$I$	$J$	$\sigma$
7.45	0.619	0.968	15.7	6.03	− 0.00106	7.97	− 0.00102	8.14	0.0248	0.0635	0.120	0.0727	5.00

### Bayesian formalization

We considered the joint probability distribution for all variables as

13 $$p\left({\varvec{D}},{{\varvec{\theta}}}_{M},M,\sigma \right)=p\left({\varvec{D}}|{{\varvec{\theta}}}_{M},M,\sigma \right)p\left({{\varvec{\theta}}}_{M}|M\right)p\left(M\right)p\left(\sigma \right).$$

The target posterior probability distribution $p\left({{\varvec{\theta}}}_{M},\sigma |{\varvec{D}},M\right)$ is expressed by using Bayes’ theorem as

14 $$p\left({{\varvec{\theta}}}_{M},\sigma |{\varvec{D}},M\right)=\frac{p\left({\varvec{D}},{{\varvec{\theta}}}_{M},M,\sigma \right)}{\iint p\left({\varvec{D}},{{\varvec{\theta}}}_{M},M,\sigma \right)d{{\varvec{\theta}}}_{M}d\sigma }=\frac{p({\varvec{D}}|{{\varvec{\theta}}}_{M},M,\sigma )p\left({{\varvec{\theta}}}_{M}|M\right)p\left(\sigma \right)}{Z(M)},$$

where the marginal likelihood is defined as $Z(M)\equiv \iint p({\varvec{D}}|{{\varvec{\theta}}}_{M},M,\sigma )p\left({{\varvec{\theta}}}_{M}|M\right)p\left(\sigma \right)d{{\varvec{\theta}}}_{M}d\sigma$.

We obtained the marginalized posterior probability distribution for a parameter of interest by marginalizing the posterior probability distribution in Eq. (14) as $p\left({\theta }_{M}^{k}|{\varvec{D}},M\right)=\iint p\left({{\varvec{\theta}}}_{M},\sigma |{\varvec{D}},M\right)d{{\varvec{\theta}}}_{M}^{\neg k}d\sigma$, where $k$ denotes the index of a specific parameter ${\theta }_{M}^{k}$. The model selection between Model 1 and Model 2 was performed on the basis of the posterior probability distribution $p(M|{\varvec{D}})$ expressed as

15 $$p\left(M|{\varvec{D}}\right)=\frac{\iint p\left({\varvec{D}},{{\varvec{\theta}}}_{M},M,\sigma \right)d{{\varvec{\theta}}}_{M}d\sigma }{\sum_{M}\iint p\left({\varvec{D}},{{\varvec{\theta}}}_{M},M,\sigma \right)d{{\varvec{\theta}}}_{M}d\sigma }=\frac{p\left(M\right)\mathrm{exp}\left\{-F(M)\right\}}{\sum_{M}p\left(M\right)\mathrm{exp}\left\{-F(M)\right\}},$$

where $F\left(M\right)$ corresponds to Bayes free energy defined by $F\left(M\right)\equiv -\mathrm{ln}Z(M)$.

### Solution using the exchange Monte Carlo method

To obtain the posterior probability distribution $p\left({{\varvec{\theta}}}_{M},\sigma |{\varvec{D}},M\right)$ and calculate the Bayes free energy $F\left(M\right)$, we used the exchange Monte Carlo method^29^. In this method, we prepare replicated probability distributions with inverse temperatures as

16 $${p}_{\beta }\left({{\varvec{\theta}}}_{M},\sigma |{\varvec{D}},M\right)\propto \mathrm{exp}\left\{-{N}_{x}{N}_{t}\beta E\left({{\varvec{\theta}}}_{M},M,\sigma \right)\right\}p\left({{\varvec{\theta}}}_{M}|M\right)p\left(\sigma \right),$$

where $\beta$ denotes an inverse temperature. The target distribution for the exchange Monte Carlo method is the joint probability distribution expressed as

17 $$p\left({\boldsymbol{\Theta }}_{1},\cdots ,{\boldsymbol{\Theta }}_{L}\right)={\prod }_{l=1}^{L}{p}_{{\beta }_{l}}\left({\boldsymbol{\Theta }}_{l}|{\varvec{D}},M\right)$$

with the different inverse temperatures $0={\beta }_{1}<\cdots <{\beta }_{L}=1$. Here, for the sake of simplicity, we combine the parameters and noise variance into a variable as $\boldsymbol{\Theta }=\left\{{{\varvec{\theta}}}_{M},\sigma \right\}$. The exchange Monte Carlo method performs the sampling from $p\left({\boldsymbol{\Theta }}_{1},\cdots ,{\boldsymbol{\Theta }}_{L}\right)$ on the basis of the following updates.

1. Sampling in each replica

The update from ${\boldsymbol{\Theta }}_{l}$ to ${\boldsymbol{\Theta }}_{l}{\prime}$ was carried out by the Metropolis algorithm^30^ following the probability ${R}_{1}=\mathrm{min}\left(1,{r}_{1}\right)$, where

18 $${r}_{1}=\frac{{p}_{{\beta }_{l}}\left({\boldsymbol{\Theta }}_{l}{\prime}|{\varvec{D}},M\right)}{{p}_{{\beta }_{l}}\left({\boldsymbol{\Theta }}_{l}|{\varvec{D}},M\right)}.$$

2. Exchange between neighboring replicas

The exchange between ${\boldsymbol{\Theta }}_{l}$ and ${\boldsymbol{\Theta }}_{l+1}$ was carried out following the probability ${R}_{2}=\mathrm{min}\left(1,{r}_{2}\right)$, where

19 $${r}_{2}=\frac{{p}_{{\beta }_{l}}\left({\boldsymbol{\Theta }}_{l+1}|{\varvec{D}},M\right){p}_{{\beta }_{l+1}}\left({\boldsymbol{\Theta }}_{l}|{\varvec{D}},M\right)}{{p}_{{\beta }_{l}}\left({\boldsymbol{\Theta }}_{l}|{\varvec{D}},M\right){p}_{{\beta }_{l+1}}\left({\boldsymbol{\Theta }}_{l+1}|{\varvec{D}},M\right)}.$$

The exchange Monte Carlo method enables escape from local optima through the exchange process and efficient achievement of the global optimal solution. The posterior probability distribution $p\left({{\varvec{\theta}}}_{M},\sigma |{\varvec{D}},M\right)$ was obtained from the sampling result corresponding to $\beta =1$. For the calculation of Bayes free energy, we consider ${z}_{M}\left(\beta \right)$ expressed as

20 $${z}_{M}\left(\beta \right)=\iint \mathrm{exp}\left\{-{N}_{x}{N}_{t}\beta E\left({{\varvec{\theta}}}_{M},M,\sigma \right)\right\}p\left({{\varvec{\theta}}}_{M}|M\right)p\left(\sigma \right)d{{\varvec{\theta}}}_{M}d\sigma .$$

Using the fact that ${z}_{M}\left(0\right)=1$, ${z}_{M}\left(1\right)$ can be calculated as follows:

21 $${z}_{M}\left(1\right)=\frac{{z}_{M}({\beta }_{L})}{{z}_{M}({\beta }_{L-1})}\frac{{z}_{M}({\beta }_{L-1})}{{z}_{M}({\beta }_{L-2})}\cdots \frac{{z}_{M}\left({\beta }_{2}\right)}{{z}_{M}\left({\beta }_{1}\right)}={\prod }_{l=1}^{L-1}\frac{{z}_{M}\left({\beta }_{l+1}\right)}{{z}_{M}\left({\beta }_{l}\right)}$$

22 $$={\prod }_{l=1}^{L-1}\frac{\iint \mathrm{exp}\left\{-{N}_{x}{N}_{t}{\beta }_{l+1}E\left({{\varvec{\theta}}}_{M},M,\sigma \right)\right\}p\left({{\varvec{\theta}}}_{M}|M\right)p\left(\sigma \right)d{{\varvec{\theta}}}_{M}d\sigma }{\iint \mathrm{exp}\left\{-{N}_{x}{N}_{t}{\beta }_{l}E\left({{\varvec{\theta}}}_{M},M,\sigma \right)\right\}p\left({{\varvec{\theta}}}_{M}|M\right)p\left(\sigma \right)d{{\varvec{\theta}}}_{M}d\sigma }$$

23 $$={\prod }_{l=1}^{L-1}{\langle \mathrm{exp}\left\{-{N}_{x}{N}_{t}({\beta }_{l+1}-{\beta }_{l})E\left({{\varvec{\theta}}}_{M},M,\sigma \right)\right\}\rangle }_{{p}_{{\beta }_{l}}\left({{\varvec{\theta}}}_{M},\sigma |{\varvec{D}},M\right)},$$

where ${\langle \cdot \rangle }_{{p}_{{\beta }_{l}}\left({{\varvec{\theta}}}_{M},\sigma |{\varvec{D}},M\right)}$ means the expectation for ${p}_{{\beta }_{l}}\left({{\varvec{\theta}}}_{M},\sigma |{\varvec{D}},M\right)$. Consequently, Bayes free energy was obtained as $F\left(M\right)=-\mathrm{ln}{z}_{M}(1)$.

### Model functions in the conventional non-linear least-squares fitting

In the fitting of the diffraction peaks at each time point (Step 1), the model function is expressed as

24 $${f}_{1}\left(x;{a}_{1},{c}_{1},{w}_{1},{a}_{2},{c}_{2},{w}_{2}\right)={\sum }_{i=1}^{2}g\left\{x;{a}_{i},{c}_{i},{w}_{i} \right\},$$

where

25 $$g\left\{x;{a}_{i},{c}_{i},{w}_{i} \right\}=\frac{\sqrt{4\mathrm{ln}2}}{\sqrt{\pi }}\frac{{a}_{i}}{{w}_{i}}\mathrm{exp}\left[-\frac{4\mathrm{ln}2{\left\{x-{c}_{i}\right\}}^{2}}{{w}_{i}^{2}}\right].$$

Here, $i=1$ and $i=2$ correspond to the adsorption and desorption phases, respectively. ${a}_{i}$, ${c}_{i}$, and ${w}_{i}$ represent the peak area, center angle, and full width at half maximum, respectively.

In the fitting of the time variation of the adsorption peak area (Step 2), we used the Kolmogorov–Johnson–Mehl–Avrami equation^23,24^ as

26 $$ f_{2} \left( {t;A,\kappa ,n} \right) = \left\{ {\begin{array}{*{20}c}    {A\left[ {1 - {\text{exp}}\left\{ { - \kappa \left( {t - t_{s} } \right)^{n} } \right\}} \right]} & {t \ge t_{s} }  \\    0 & {t < t_{{s,}} }  \\   \end{array} } \right. $$

where $A$, $\kappa$, and $n$ indicate the scale parameter, rate constant, and number of dimensions, respectively, while ${t}_{s}$ denotes the gas-shot time and is determined by information from the apparatus.

## Data Availability

The datasets used and/or analysed during the current study available from the corresponding author on reasonable request.
